# An Elderly Patient With Status Epilepticus Caused by a Dural Arteriovenous Fistula

**DOI:** 10.7759/cureus.109568

**Published:** 2026-05-24

**Authors:** Takuya Saito, Hiroshi Fukaya, Shunsuke Omodaka, Yukako Yazawa

**Affiliations:** 1 Department of Stroke Neurology, Kohnan Hospital, Sendai, JPN; 2 Department of Neuroendovascular Therapy, Kohnan Hospital, Sendai, JPN

**Keywords:** alzheimer's disease, dural arteriovenous fistulas, neurology, neurosurgery, radiology

## Abstract

Dural arteriovenous fistulas (DAVFs) are a rare cause of epilepsy. Therefore, they can be misdiagnosed or overlooked. We report a case of an 84-year-old female with Alzheimer's disease who developed status epilepticus as the initial manifestation of the left transverse sinus DAVF. On admission, symptomatic epilepsy secondary to Alzheimer’s disease was suspected, and medical therapy was started; however, clinical improvement was limited. Magnetic resonance imaging and magnetic resonance angiography findings provided clues for the diagnosis of the left transverse sinus DAVF. Endovascular treatment led to improvement in behavioral symptoms and electroencephalography findings with no seizure recurrence. One month later, she returned to her baseline condition. This case highlights the importance of including DAVF in the differential diagnosis of seizures in older adults and of recognizing that invasive endovascular therapy may offer a curative option.

## Introduction

The incidence of epilepsy is increasing in older adults [1]. The most common findings on brain magnetic resonance imaging (MRI) in older adult patients with epilepsy are stroke and brain atrophy [2]. Definitive treatment depends on the underlying etiology; however, antiseizure medications are the mainstay of epilepsy treatment, and up to two-thirds of patients may experience seizure suppression with medication [3]. Dural arteriovenous fistulas (DAVFs) are rare, but their incidence is rising with aging [4]. Seizure can occasionally be the presenting symptom of DAVF [5]. Herein, we present a rare case of an older female with status epilepticus caused by a DAVF, who achieved complete neurological recovery and remained seizure-free during follow-up following endovascular treatment.

## Case presentation

An 84-year-old female with a history of mild Alzheimer’s disease and no prior seizure history presented with new-onset focal-onset seizures. She developed recurrent clonic jerks involving the right lower face and right upper limb with preserved awareness, consistent with a focal aware motor seizure, and was admitted to our hospital. She presented with rightward forced gaze deviation and ongoing focal impaired awareness motor seizures with clonic activity involving the right face and right upper limb. Diazepam 5 mg and fosphenytoin 22.5 mg/kg were administered intravenously, and the seizures ceased. MRI showed high signal intensity in the left insular cortex, temporal lobe, and thalamus on diffusion-weighted imaging, and a small hypointense lesion in the left temporal lobe on T2*-weighted imaging (Figure 1). Blood test results were unremarkable. Cerebrospinal fluid examination revealed a cell count of 5 cells/μL and a protein level of 99 mg/dL.

**Figure 1:**
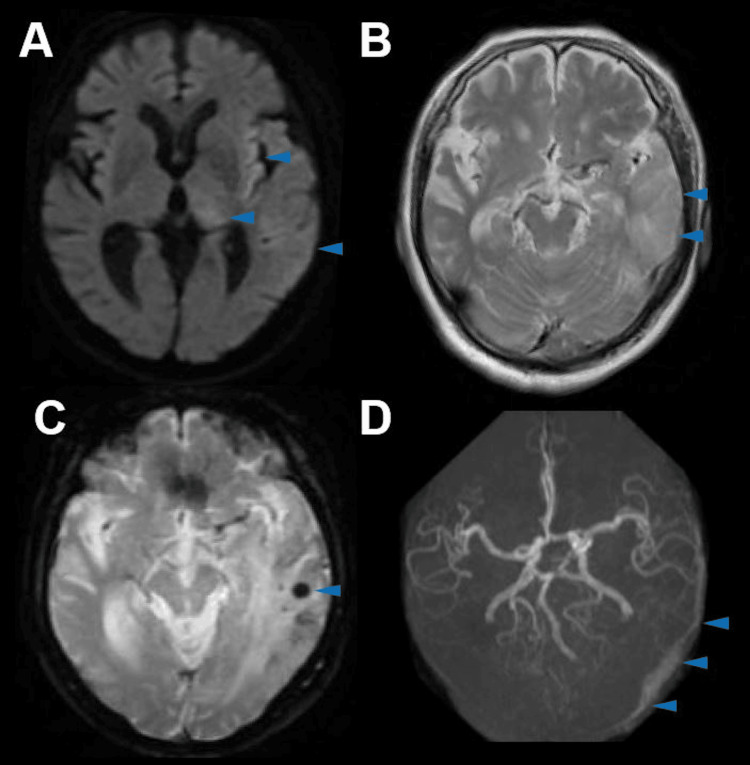
Magnetic resonance imaging findings at admission. (A) Axial diffusion-weighted imaging shows high signal intensity in the left insular cortex, temporal lobe, and thalamus (arrowheads). (B) Axial T2-weighted imaging shows hyperintensity in the left temporal lobe (arrowheads). (C) Axial T2*-weighted gradient-recalled echo imaging shows a small hypointense lesion in the left temporal lobe (arrowhead). (D) Time-of-flight magnetic resonance angiography shows visualization of the left transverse sinus (arrowheads).

The patient was diagnosed with status epilepticus and was treated with levetiracetam 1,000 mg/day. Although no further seizures occurred and her consciousness improved, she remained irritable, restless, and agitated, symptoms that were uncharacteristic of her premorbid personality. Electroencephalography (EEG) showed diffuse slowing with left hemispheric predominance, along with frequent recurrent sharp waves maximal over the left frontal region, and non-convulsive status epilepticus (NCSE) was suspected. Increasing the levetiracetam dose and adding lacosamide failed to improve the EEG findings or neuropsychiatric symptoms.

On day 10, follow-up MRI revealed marked edema in the left temporal lobe. Magnetic resonance angiography (MRA) raised suspicion of DAVF. Cerebral angiography confirmed DAVF of the left transverse-sigmoid sinus with cortical venous reflux (CVR) (Borden type II, Cognard type IIb) (Figure 2) [2]. The patient underwent transarterial and transvenous embolization, which resulted in complete obliteration of the shunt and CVR (Figure 3).

**Figure 2:**
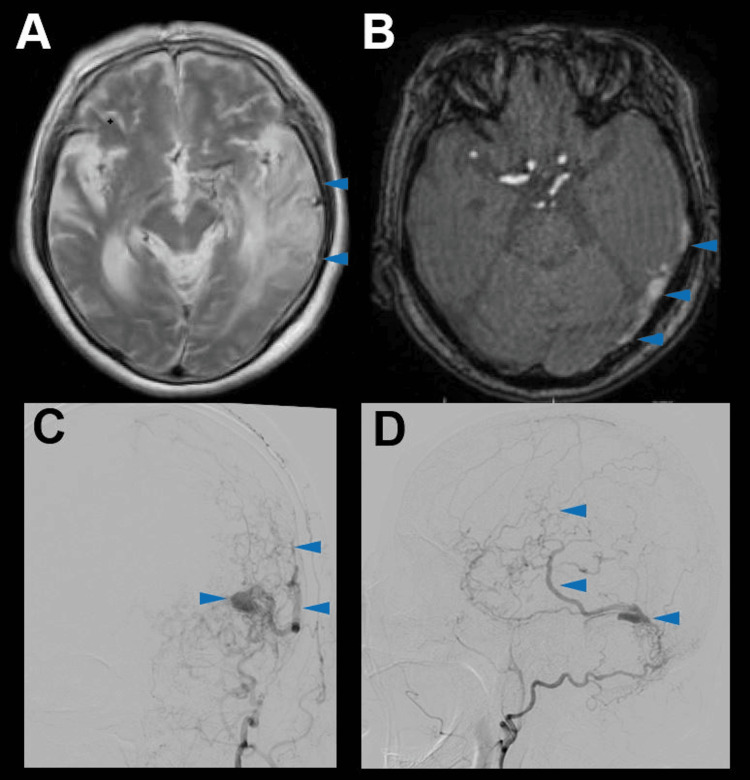
Magnetic resonance imaging and cerebral angiography findings after admission. (A) Axial T2-weighted imaging shows marked edematous changes in the left temporal lobe (arrowheads). (B) Time-of-flight magnetic resonance angiography shows abnormal cerebral veins (arrowheads). (C) Digital subtraction angiography of the left external carotid artery (anteroposterior view) shows a dural arteriovenous fistula at the left transverse–sigmoid sinus with cortical venous reflux (arrowheads). (D) The same angiography (lateral view) shows a dural arteriovenous fistula at the left transverse–sigmoid sinus with cortical venous reflux (arrowheads).

**Figure 3:**
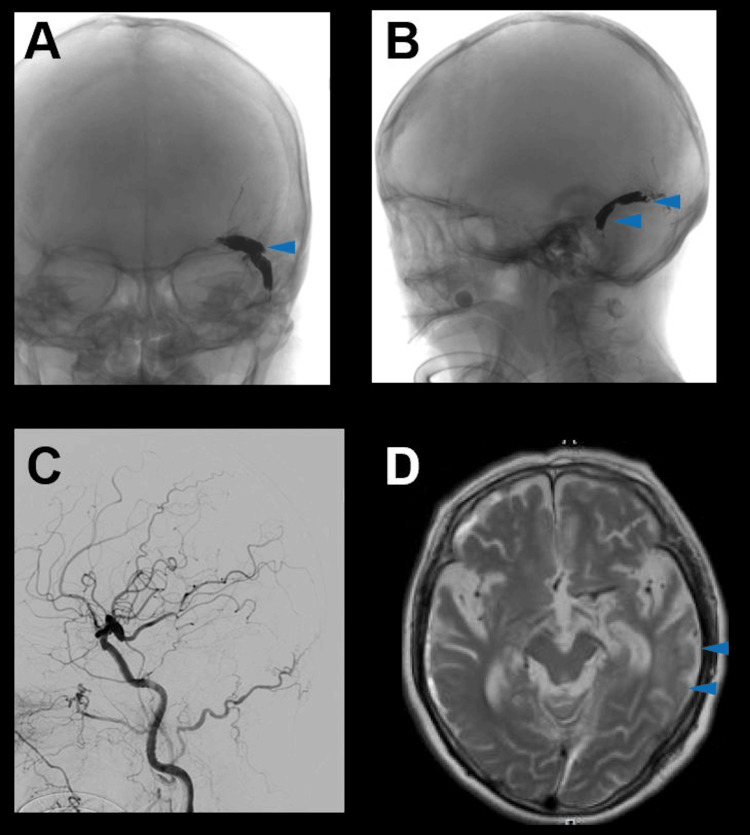
Cerebral angiography and magnetic resonance imaging findings after endovascular therapy. (A) Unsubtracted fluoroscopic image (anteroposterior view) shows embolization materials and coils placed within the dural venous sinuses and cortical veins (arrowhead indicates the treated left transverse sinus). (B) Unsubtracted fluoroscopic image (lateral view) shows embolization materials and coils placed within the dural venous sinuses and cortical veins (arrowheads indicate the treated left transverse sinus). (C) Digital subtraction angiography of the left common carotid artery (lateral view) shows complete obliteration of the shunt with disappearance of cortical venous reflux. (D) Axial T2-weighted imaging shows resolution of the left temporal lobe edema (arrowheads).

Post-treatment MRI showed resolution of the brain edema (Figure 3). Her neuropsychiatric symptoms and EEG findings gradually improved. One month later, she returned to her premorbid calm demeanor.

## Discussion

We report the case of an older patient who developed status epilepticus. The seizures were attributed to epilepsy secondary to Alzheimer's disease, and psychiatric symptoms such as agitation were incorrectly attributed to the behavioral and psychological symptoms of Alzheimer's disease. In the differential diagnosis, infectious or autoimmune encephalitis was considered but deemed unlikely based on the cerebrospinal fluid findings. Head trauma was not suspected from the clinical history. Cerebral venous sinus thrombosis and intracranial neoplasm were also considered; however, these were excluded on the basis of MRI and cerebral angiography findings. Finally, the EEG and MRI findings led to the correct diagnosis of DAVF. Endovascular embolization successfully cured the seizures and resolved the associated neuropsychiatric symptoms. To the best of our knowledge, reports on older patients with DAVF who developed seizures are scarce, making this case report informative.

DAVF is a rare condition that is often overlooked, yet should be considered in the diagnosis of seizures. CVR may generate retrograde venous flow that disrupts the blood-brain barrier and promotes leakage of serum-derived components, thereby increasing cortical irritability and contributing to regional epileptogenicity [5,6]. In this case, worsening brain edema on MRI and abnormal flow on MRA were the key diagnostic clues. Endovascular embolization is the standard treatment for DAVFs. A systematic review of DAVF-related seizures indicated that antiseizure medications alone are often insufficient, whereas endovascular therapy significantly improves clinical outcomes [6]. Although invasive procedures are often avoided in older adult patients, curative treatment should be considered in patients with DAVF-related seizures.

The main limitation of this report is that it describes only a single case, and the findings cannot be generalized. Although this patient had no treatment-related complications, the risks of endovascular therapy must be weighed against its benefits, particularly in older adults [7].

## Conclusions

We report the case of an older adult patient with DAVF-related seizures and NCSE, who was successfully treated with endovascular embolization. In this case, changes in MRI and MRA findings were essential for diagnosis. Clinicians should consider DAVF in the differential diagnosis of new-onset seizures in older adult patients, and endovascular treatment should be considered, even in older individuals, when curative potential exists.
